# Antagonistic effects of IL-17 and D-resolvins on endothelial Del-1 expression through a GSK-3β-C/EBPβ pathway

**DOI:** 10.1038/ncomms9272

**Published:** 2015-09-16

**Authors:** Tomoki Maekawa, Kavita Hosur, Toshiharu Abe, Alpdogan Kantarci, Athanasios Ziogas, Baomei Wang, Thomas E. Van Dyke, Triantafyllos Chavakis, George Hajishengallis

**Affiliations:** 1 Department of Microbiology, Penn Dental Medicine, University of Pennsylvania, 240 S. 40th Street, Philadelphia, Pennsylvania 19104, USA; 2 Niigata University, Graduate School of Medical and Dental Sciences, Research Center for Advanced Oral Science, 2-5274 Gakkocho-dori, Chuo-ku, Niigata 951-8514, Japan; 3 Department of Applied Oral Sciences, Center for Periodontology, The Forsyth Institute, 245 First Street, Cambridge, Massachusetts 02142, USA; 4 Department of Clinical Pathobiochemistry and Institute for Clinical Chemistry and Laboratory Medicine, Technische Universität Dresden, Fetscherstraße 74, Dresden 01307, Germany

## Abstract

Del-1 is an endothelial cell-secreted anti-inflammatory protein. In humans and mice, Del-1 expression is inversely related to that of IL-17, which inhibits Del-1 through hitherto unidentified mechanism(s). Here we show that IL-17 downregulates human endothelial cell expression of Del-1 by targeting a critical transcription factor, C/EBPβ. Specifically, IL-17 causes GSK-3β-dependent phosphorylation of C/EBPβ, which is associated with diminished C/EBPβ binding to the Del-1 promoter and suppressed Del-1 expression. This inhibitory action of IL-17 can be reversed at the GSK-3β level by PI3K/Akt signalling induced by D-resolvins. The biological relevance of this regulatory network is confirmed in a mouse model of inflammatory periodontitis. Intriguingly, resolvin-D1 (RvD1) confers protection against IL-17-driven periodontal bone loss in a Del-1-dependent manner, indicating an RvD1-Del-1 axis against IL-17-induced pathological inflammation. The dissection of signalling pathways regulating Del-1 expression provides potential targets to treat inflammatory diseases associated with diminished Del-1 expression, such as periodontitis and multiple sclerosis.

## Abstract (web summary)

The endothelial secreted protein Del-1 plays a role in limiting inflammation, and its deficiency is associated with pathology in periodontitis and multiple sclerosis. Here the authors show that the negative regulation of Del-1 by IL-17 involves targeting the transcription factor C/EBPß in a GSK-3ß- dependent manner.

## Graphical abstract

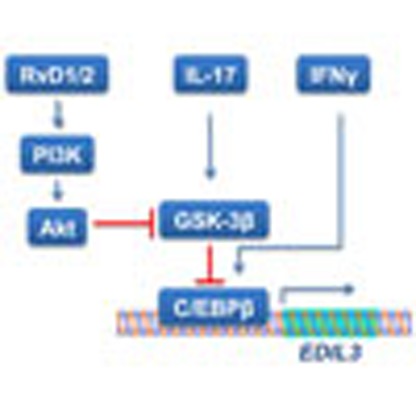

The proper function of homeostatic mechanisms is essential for protection against unwarranted inflammatory tissue damage^1^,^2^. In this regard, developmental endothelial locus 1 (Del-1), an endothelial cell-secreted protein, acts homoeostatically to suppress inflammation in various organs and tissues, including mucosal sites and the central nervous system^3^,^4^,^5^,^6^,^7^. Mechanistically, Del-1 interferes with β_2_ integrin-dependent inflammatory cell adhesion to the endothelium and thereby restrains neutrophil transendothelial migration^3^,^4^,^8^. Genetic deficiency of Del-1 not only leads to excessive neutrophil infiltration but also causes interleukin (IL)-17-dependent inflammatory tissue damage in mouse models of periodontitis^4^ and multiple sclerosis^5^. Conversely, inactivating IL-17 signalling in Del-1-deficient mice, that is, in mice with combined deficiency in Del-1 and the IL-17 receptor, reverses the Del-1 deficiency-related inflammatory disorders^4^,^5^.

IL-17 signal transduction regulates a variety of pro-inflammatory and antimicrobial genes by activating NF-κB and mitogen-activated protein kinase (MAPK) pathways as well as the CCAAT/enhancer-binding protein (C/EBP) family of transcription factors, such as C/EBPβ and C/EBPδ (ref. 9). These IL-17 receptor (IL-17RA/RC) signalling events are mediated through a conserved SEF/IL-17R (SEFIR) domain that engages the Act1 adaptor protein. On the other hand, the distal C/EBPβ activation domain (CBAD) of IL-17RA coordinates inhibitory signals. For instance, CBAD is required for glycogen synthase kinase-3β (GSK-3β)-mediated inhibitory phosphorylation of C/EBPβ that suppresses its transcriptional activity^9^,^10^. It has long been established that IL-17 promotes neutrophil recruitment through the induction of CXC chemokines (for example, CXCL1, CXCL2 and CXCL8)^11^. More recently, we have shown that IL-17 downregulates the expression of Del-1 in endothelial cells, thereby mitigating the antagonistic effect of Del-1 on neutrophil extravasation^4^. The ability of IL-17 to inhibit Del-1—and hence facilitate neutrophil recruitment—may be beneficial in the context of an acute response to pathogen infection or in oropharyngeal candidiasis, where neutrophils play a crucial role in host defense^12^. However, chronic low levels of Del-1 could potentially contribute to inflammatory disease pathology. This notion is consistent with our detection of significantly decreased Del-1 expression in biopsies from patients with periodontitis^4^ or multiple sclerosis^5^ relative to healthy tissue specimens. Intriguingly, Del-1 expression is diminished in brain tissue from patients with chronic-active multiple sclerosis lesions^5^, further suggesting that low Del-1 levels are associated with disease activity. Similarly to observations in humans, Del-1 expression is severely decreased in the periodontal tissue of aged mice with naturally occurring periodontitis^4^ and in the spinal cords of mice with experimental autoimmune encephalomyelitis, the rodent equivalent of multiple sclerosis^5^. Therefore, mice appear to constitute an appropriate model to study the regulation of Del-1 expression.

Since the expression levels of Del-1 appear to determine disease activity in at least some IL-17-associated inflammatory disorders, the elucidation of the mechanisms and factors that regulate Del-1 expression may have significant therapeutic value. However, very little is currently known about the regulation of Del-1 expression. The main objective of this study was therefore to understand how IL-17 inhibits Del-1 expression and identify mechanisms that can reverse this inhibition. In this regard, we hypothesized that molecules involved in the resolution of inflammation can interfere with IL-17-mediated inhibition of Del-1 expression. Resolution of inflammation is now appreciated to be a well-coordinated active process aiming to reinstate tissue integrity and function and is mediated by specialized pro-resolving lipid mediators that act through specific receptors^13^. The specialized pro-resolving agonists include arachidonic acid-derived lipoxins and omega-3 polyunsaturated fatty acid-derived resolvins and protectins^14^,^15^,^16^. Since the resolution of inflammation entails termination of unwarranted neutrophil recruitment and Del-1 is a potent inhibitor of neutrophil transmigration, we hypothesized that pro-resolving molecules could restore the expression of Del-1. Here we show that IL-17 downregulates Del-1 expression in human umbilical vein endothelial cells (HUVEC) through a GSK-3β- and C/EBPβ-dependent pathway. Importantly, the inhibitory action of IL-17 on Del-1 expression is counteracted by resolvins D1 and D2 at the GSK-3β level in a phosphatidylinositol 3-kinase (PI3K)/Akt-dependent manner. The biological relevance of these regulatory interactions is confirmed *in vivo*, thereby providing novel potential therapeutic targets to treat inflammatory disorders associated with diminished Del-1 expression.

## Results

### GSK-3β and C/EBPβ are involved in IL-17 regulation of Del-1

**Figure 1:**
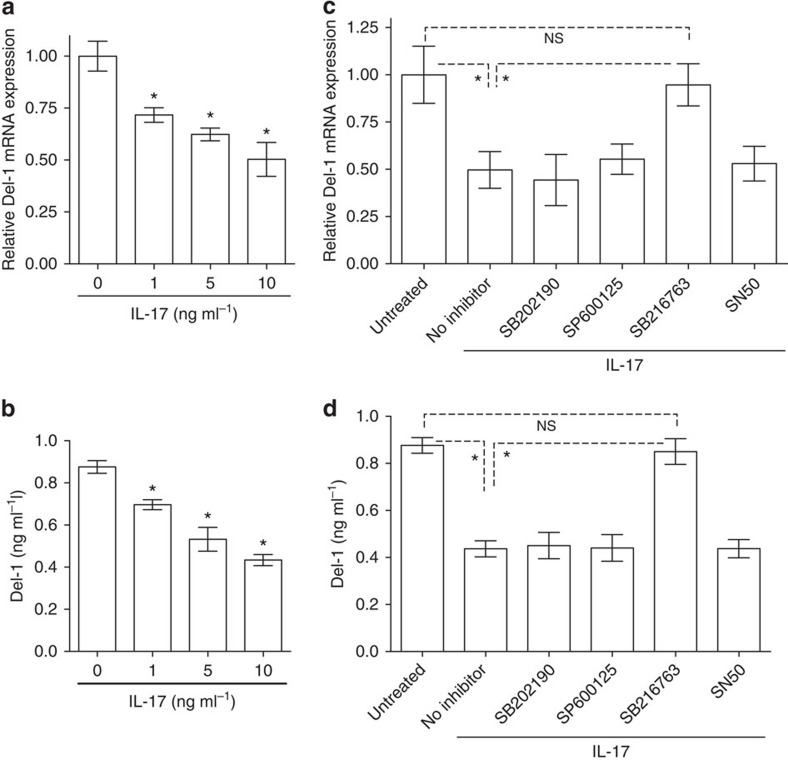
Inhibition of GSK-3β reverses the inhibitory effect of IL-17 on Del-1 expression. (**a**) Del-1 mRNA expression in HUVEC treated for 2 h with the indicated concentrations of IL-17. (**b**) Similar experimental setup as above for ELISA determination of Del-1 protein levels in culture supernatants after 6 h incubation. (**c**) HUVEC were stimulated or not with IL-17 (5 ng ml^−1^; 2 h incubation), in the absence or presence of SB202190 (20 μM; p38 MAPK inhibitor), SP600125 (50 μM; JNK inhibitor), SB216763 (10 μM; GSK-3β inhibitor) or SN50 (50 μM; NF-κB inhibitor). Del-1 mRNA expression was determined by qPCR. Results were normalized to those of GAPDH mRNA and expressed as fold change in transcript levels relative to those of untreated control (medium only), the average value of which was taken as 1. (**d**) Similar experimental setup as in (**c**) was used for ELISA determination of Del-1 protein levels in culture supernatants after 6 h incubation. Data are means±s.d. (**a**,**c** *n*=3 and **b**,**d** *n*=5 sets of HUVEC cultures). *P<0.01 compared to control or between indicated groups (ANOVA). NS, not significant.

We have previously shown that gingival Del-1 expression is downregulated by IL-17 receptor signalling on endothelial cells, resulting in enhanced recruitment of neutrophils to the murine gingival tissue^4^. Moreover, IL-17 inhibits endothelial Del-1 expression in HUVEC^4^, a finding that was confirmed here in a dose-dependent manner at both the mRNA (Fig. 1a) and protein (Fig. 1b) levels. Therefore, HUVEC constitute a relevant model to study Del-1 regulation by IL-17. As a first step to dissect the pathway of IL-17-induced inhibition of Del-1 expression, we investigated possible involvement of molecules activated downstream of the IL-17 receptor complex^11^, namely, NF-κB and major kinases, p38 MAPK, JNK and GSK-3β. Pharmacological inhibition of GSK-3β with SB216763 (but not of the other investigated molecules by appropriate inhibitors) significantly reversed the IL-17 inhibitory effect on Del-1 mRNA and protein expression (Fig. 1c,d, respectively). Independent confirmation of this finding was obtained by silencing GSK-3β (Supplementary Fig. 1a). In Fig. 1 and throughout the study, SB216763 was used at 10 μM, which was the minimum concentration yielding maximum inhibition of IL-17-mediated suppression of Del-1 expression (Supplementary Fig. 1b; see Supplementary Fig. 1c–g for detailed dose–response experiments of other inhibitors and short-interfering RNA (siRNA) used in the study). Although GSK-3β has many potential targets in general^17^,^18^, in IL-17-stimulated cells GSK-3β was shown to phosphorylate the transcription factor C/EBPβ, leading to inhibition of its transcriptional activity^10^. In contrast to IL-17, IFNγ enhances the transcriptional activity of C/EBPβ (refs 19, 20) and, moreover, upregulates Del-1 expression in HUVEC^21^. Taken together, these findings suggested that C/EBPβ may be involved in the regulation of Del-1 expression. In this regard, *in silico* promoter analysis using TRANSFAC (v. 2014.4) suggested the presence of two putative binding sites for C/EBPβ in the promoter region of the Del-1-encoding gene (*EDIL3*) that were confirmed by chromatin immunoprecipitation (ChIP)-seq analysis (according to TRANSFAC database; http://www.biobase-international.com).

**Figure 2:**
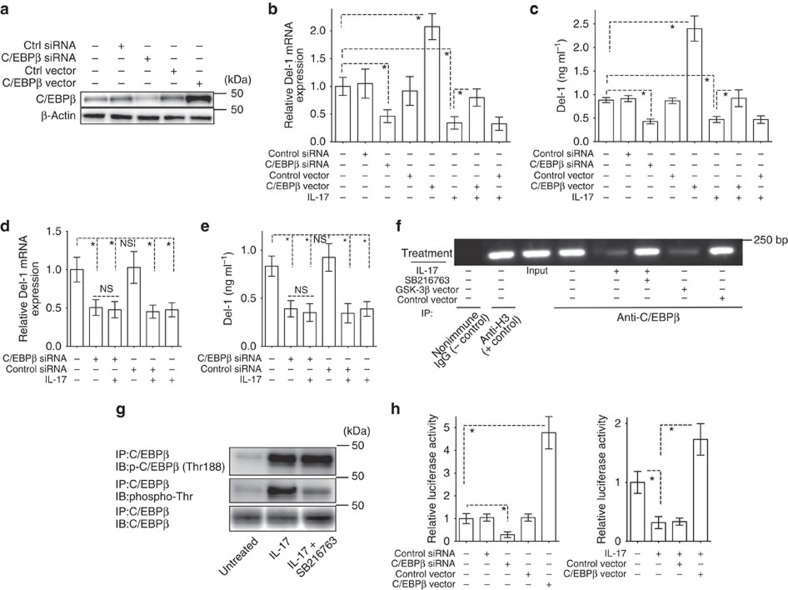
C/EBPβ regulates Del-1 expression in HUVEC. HUVEC were transfected with control siRNA, siRNA to C/EBPβ, control vector or with C/EBPβ expression vector. (**a**) After 24 h, the cells were lysed and whole-cell-lysate immunoblotting with specific antibodies was used to monitor the levels of C/EBPβ protein (β-actin was used as control). (**b**,**c**) 24 h after transfection, IL-17 (5 ng ml^−1^) was added to the cells for 2 or 6 h incubation to determine, respectively, Del-1 mRNA expression by qPCR (**a**) or Del-1 protein levels in culture supernatants by ELISA (**c**). The qPCR results were normalized to those of GAPDH mRNA and expressed as fold change in transcript levels relative to those of untreated control, the average value of which was taken as 1. (**d**,**e**). In a similar experimental setup as in (**b**,**c**) HUVEC were treated as indicated and processed for determination of Del-1 mRNA expression by qPCR (**d**) or Del-1 protein levels in culture supernatants by ELISA (**e**). (**f**) ChIP analysis of C/EBPβ binding to *EDIL3* promoter in HUVEC which were treated or not with IL-17 (10 ng ml^−1^; 4 h) with or without 1-h pre-treatment with SB216763 (10 μM; GSK-3β inhibitor), or were transfected with GSK-3β expression vector or empty control vector. IP, immunoprecipitation. (**g**) HUVEC were treated or not with IL-17 (10 ng ml^−1^; 30 min), in the presence of SB216763 (10 μM; GSK3β inhibitor) which was added 1 h earlier than IL-17. Cell lysates immunoprecipitated using anti-C/EBPβ were immunoblotted with anti-phospho-C/EBPβ (Thr-188), anti-phospho-threonine antibody, or with anti-C/EBPβ (control). (**h**) HEK-293T cells were transfected with a hEDIL3-promoter-Luc reporter plasmid along with siRNA to C/EBP (or control siRNA), or with a C/EBPβ expression vector (or control vector) and analysed for luciferase activity after 16 h (left panel). HEK-293T-IL-17RA/C cells were transfected with hEDIL3-promoter-Luc reporter plasmid and C/EBPβ expression vector (or control vector) in the absence or presence of IL-17 (10 ng ml^−1^) and analysed for luciferase activity after 16 h (right panel). Data are means±s.d. (**b**–**e**, *n*=5; **h**, *n*=4 sets of HUVEC cultures). *P<0.01 between indicated groups (ANOVA).

To test the hypothesis that C/EBPβ regulates Del-1, we performed siRNA-mediated silencing of the *C/EBPβ* gene in HUVEC and confirmed diminished expression of C/EBPβ protein (Fig. 2a). Importantly, the treatment with siRNA to C/EBPβ (but not with control siRNA) also reduced the basal expression of Del-1 at the protein and mRNA level (Fig. 2b,c). Conversely, overexpression of C/EBPβ in HUVEC enhanced the basal expression of Del-1 (Fig. 2b,c). Moreover, the ability of IL-17 to inhibit Del-1 mRNA and protein expression was counteracted by overexpression of C/EBPβ (Fig. 2b,c; next-to-last bar). In C/EBPβ-knocked-down HUVEC, IL-17 failed to cause further inhibition of Del-1 mRNA and protein expression (Fig. 2d,e), suggesting that IL-17 does not mediate C/EBPβ-independent effects in Del-1 regulation.

To further understand the mechanism by which IL-17 regulates Del-1, we performed ChIP analysis in HUVEC. IL-17 treatment of HUVEC resulted in reduced occupancy of C/EBPβ at the promoter region of the gene encoding Del-1 (*EDIL3;* EGF-like repeats and discoidin I-like domains 3), relative to untreated cells (Fig. 2f). However, the ability of IL-17 to inhibit the recruitment of C/EBPβ to the *EDIL3* promoter region was reversed by SB216763, indicating that the inhibitory effect of IL-17 is mediated by GSK-3β (Fig. 2f). Consistently, overexpression of GSK-3β in HUVEC (confirmed by western blotting; Supplementary Fig. 2) led to diminished occupancy of C/EBPβ at the *EDIL3* promoter region relative to cells transfected with control vector (Fig. 2f, last two lanes). Therefore, GSK-3β regulates the recruitment of C/EBPβ to the *EDIL3* promoter.

We thus next examined whether the ability of the GSK-3β inhibitor, SB216763, to restore the binding of C/EBPβ to the *EDIL3* promoter in IL-17-treated HUVEC (Fig. 2f) involves changes in C/EBPβ phosphorylation. To this end, C/EBPβ was immunoprecipitated by specific antibody from lysates of HUVEC pretreated with IL-17 in the absence or presence of SB216763. The immunoprecipitated protein was subjected to immunoblotting and probed with anti-phospho-Thr antibody or with anti-phospho-C/EBPβ-Thr188 (negative control, as the IL-17-induced phosphorylation of this residue is GSK-3β-independent^10^). As expected, SB216763 did not influence IL-17-induced phosphorylation of C/EBPβ at Thr-188 (Fig. 2g, upper panel). Importantly, however, SB216763 greatly diminished the overall Thr phosphorylation of C/EBPβ (Fig. 2g, middle panel). Taken together with the earlier data on IL-17-treated HUVEC, it can be inferred that GSK-3β-dependent phosphorylation of C/EBPβ is associated with diminished C/EBPβ binding to the *EDIL3* promoter and suppressed Del-1 expression.

To further substantiate that Del-1 is a target of C/EBPβ, we subcloned the Del-1 (EDIL3) promoter region into a luciferase reporter plasmid. Co-transfection of HEK-293T cells with the reporter plasmid (hEDIL3-promoter-Luc) and siRNA to C/EBPβ resulted in decreased luciferase activity compared with co-transfection with control siRNA (Fig. 2h, left). Conversely, and consistently, co-transfection of HEK-293T cells with hEDIL3-promoter-Luc and a C/EBPβ expression vector resulted in increased luciferase activity compared to co-transfection with a control vector (Fig. 2h, left). Moreover, the luciferase activity of hEDIL3-promoter-Luc-transfected HEK-293T-IL-17RA/C cells was inhibited by IL-17, and this inhibitory effect was counteracted by overexpressing C/EBPβ (Fig. 2h, right). Although C/EBPβ can exist in three isoforms because of alternative translation (liver-enriched activating protein (LAP), LAP*, and liver-enriched inhibitory protein (LIP))^22^, IL-17 is unlikely to regulate Del-1 expression through alternative translation of *C/EBPβ*, since IL-17 treatment of HUVEC did not change the relative expression of the activating (LAP and LAP*) isoforms versus the inhibitory (LIP) isoform of C/EBPβ (Supplementary Fig. 3). Taken together, the findings in Figs 1 and 2 show that IL-17 downregulates Del-1 expression in a GSK3β- and C/EBPβ-dependent manner.

### Resolvins reverse the inhibitory action of IL-17 on Del-1

**Figure 3:**
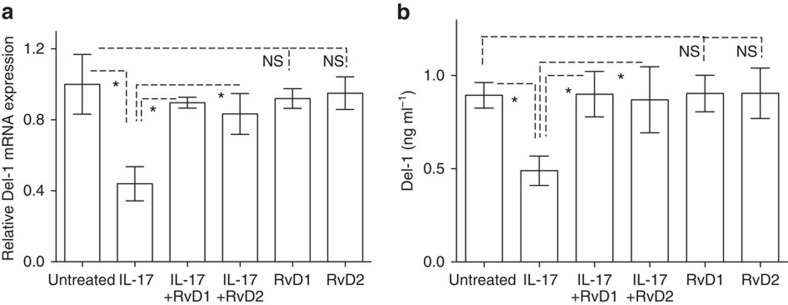
Resolvins counteract the inhibitory effect of IL-17 on Del-1 expression in HUVEC. HUVEC were stimulated as indicated (IL-17, 5 ng ml^−1^; RvD1, 100 nM; RvD2, 100 nM) for 2 h (**a**) or 6 h (**b**). Del-1 expression was determined at the mRNA level by qPCR (**a**) and at the protein level by ELISA of culture supernatants (**b**). The untreated controls represent incubation with medium only; in the qPCR assay, this group was assigned an average value of 1. Data are means±s.d. (**a**, *n*=3 and **b**, *n*=5 sets of HUVEC cultures). *P<0.01 between indicated groups (ANOVA). NS, not significant.

**Figure 4:**
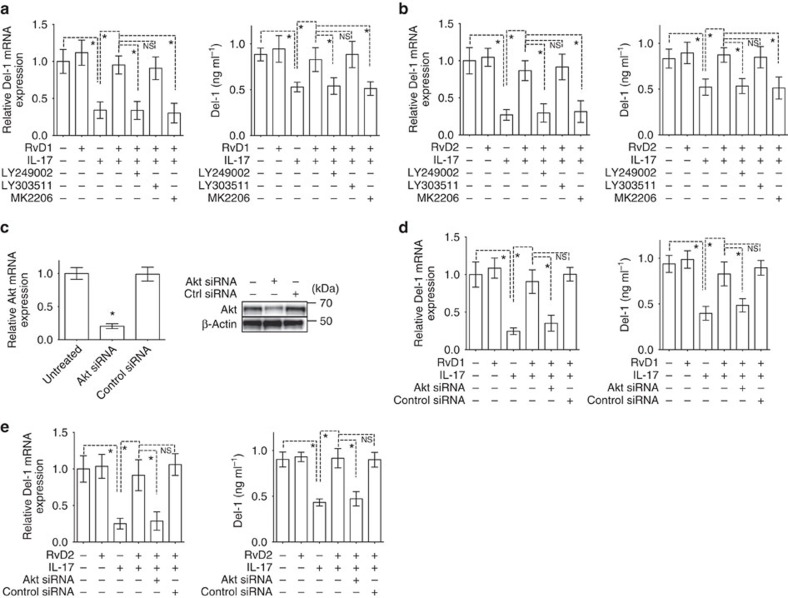
RvD1 and RvD2 reverse the inhibitory effect of IL-17 on Del-1 expression in a PI3K/Akt-dependent manner. (**a**,**b**) HUVEC were stimulated for 2 h (to determine Del-1 mRNA expression; left panels) or 6 h (to determine Del-1 protein levels; right panels) in the absence or presence of IL-17 (5 ng ml^−1^). Prior to IL-17 stimulation, the cells were pretreated for 30 min with 100 nM RvD1 (**a**) or RvD2 (**b**). In experiments using LY249002 (20 μM; PI3K inhibitor) or LY303511 (20 μM, inactive analogue), or MK2206 (Akt inhibitor; 10 μM), these compounds were added 1 h before RvD1 or RvD2. Del-1 mRNA expression was determined by qPCR. (**c**–**e**) Similar experiments as above with the exception that, in lieu of Akt inhibitor, HUVEC were treated with control or Akt-specific siRNA which effectively inhibited expression of Akt mRNA and protein (upper and lower panel, respectively, in **c**). Del-1 mRNA expression was determined by qPCR and results were normalized to those of GAPDH mRNA and expressed as fold change in transcript levels relative to those of medium only-treated cells, the average value of which was taken as 1. Del-1 protein levels in culture supernatants were determined by ELISA. Data are means±s.d. (*n*=5 sets of HUVEC cultures). *P<0.01 between indicated groups (ANOVA). NS, not significant.

The successful resolution of inflammation requires mechanisms to prevent unwarranted neutrophil infiltration. We hypothesized that resolvins can reverse the inhibitory effect of IL-17 on Del-1 expression, thereby reinstating the capacity of Del-1 to control neutrophil recruitment. Indeed, at a concentration of 100 nM (determined in dose–response experiments; Supplementary Fig. 4a,b), both resolvin-D1 (RvD1) and RvD2 completely counteracted IL-17 inhibition of Del-1 mRNA and protein expression, although they did not directly influence Del-1 expression (Fig. 3a,b). It was previously shown that Akt inhibits GSK-3β and mitigates its negative control of C/EBPβ activity, thereby restoring the DNA-binding capacity of C/EBPβ^23^. We thus hypothesized that the ability of resolvins to induce PI3K-dependent activation of Akt^24^,^25^ may underlie their capacity to antagonize IL-17. Upon confirming that RvD1 and RvD2 activate Akt (Supplementary Fig. 4c,d), we tested the hypothesis. Specifically, we investigated whether RvD1 and RvD2 lose their ability to reverse the inhibitory effect of IL-17 on Del-1 expression in HUVEC treated with LY294002 (PI3K inhibitor), LY303511 (inactive analogue control), or MK2206 (Akt inhibitor). We found that PI3K or Akt inhibition abrogated the ability of both RvD1 and RvD2 to rescue Del-1 mRNA and protein expression in IL-17-treated HUVEC (Fig. 4a,b, respectively). To conclusively confirm the involvement of Akt in the pathway activated by the resolvins, we silenced Akt expression by specific siRNA treatment (Fig. 4c). Consistent with the findings of the pharmacological inhibition experiment using MK2206, Akt knockdown abrogated the capacity of RvD1 or RvD2 to restore Del-1 expression in IL-17-treated HUVEC (Fig. 4d,e, respectively).

**Figure 5:**
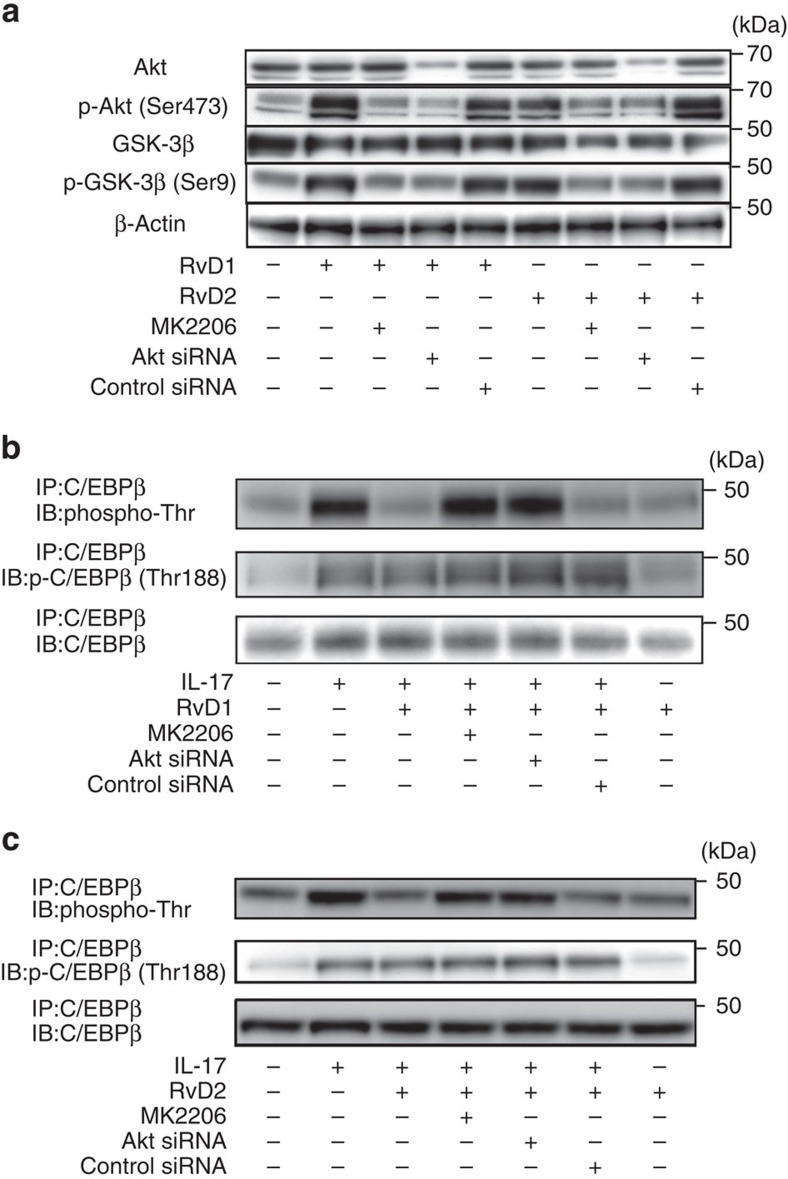
RvD1 and RvD2 induce Ser9 phosphorylation of GSK-3β and inhibit IL-17-induced phosphorylation of C/EBPβ in an Akt-dependent way. (**a**) HUVEC were pretreated with MK2206 for 1 h, or with control siRNA or specific siRNA to Akt (20 nM) for 24 h, and then incubated with or without RvD1 or RvD2 (both at 100 nM). After 15 min, the phosphorylation of Akt (Ser473) or GSK-3β (Ser9) was determined by immunoblotting with specific phospho-antibodies. (**b**) HUVEC were pretreated with MK2206 for 1 h, or with control siRNA or specific siRNA to Akt (20 nM) for 24 h. The cells were then incubated with IL-17 (5 ng ml^−1^) for 30 min in the absence of presence of RvD1 (100 nM). Cell lysates immunoprecipitated using anti-C/EBPβ were immunoblotted with anti-phospho-C/EBPβ (Thr-188), anti-phospho-threonine antibody, or with anti-C/EBPβ (control). (**c**) Similar experiment as in (**b**) using RvD2 in lieu of RvD1.

In IL-17-treated HUVEC, GSK-3β-dependent phosphorylation of C/EBPβ is associated with diminished C/EBPβ binding to the *EDIL3* promoter and inhibition of Del-1 expression (Fig. 2). Since RvD1 and RvD2 restored Del-1 expression in IL-17-treated HUVEC, it is possible that they target this GSK-3β-C/EBPβ mechanism. To explore this possibility, we first showed that RvD1 and RvD2 induce Ser9 phosphorylation of GSK-3β in an Akt-dependent way (Fig. 5a). Since Ser9 phosphorylation of GSK-3β inhibits the activity of this kinase^26^, we hypothesized that RvD1 and RvD2 can block the GSK-3β-dependent phosphorylation of C/EBPβ in IL-17-treated cells (Fig. 2g). Indeed, we then showed that RvD1 and RvD2 inhibited IL-17-induced phosphorylation of C/EBPβ in a Akt-dependent manner, although—as expected—they failed to affect C/EBPβ phosphorylation at Thr188 which is GSK-3β-independent^10^ (Fig. 5b,c). These data suggest that RvD1 and RvD2 can promote the transcriptional activity of C/EBPβ in IL-17-treated cells, which is consistent with their ability to upregulate the C/EBPβ-dependent (Fig. 2b–e,h) expression of Del-1 (Fig. 3).

### GPR32 and ALX/FPR2 mediate resolvin regulation of Del-1

**Figure 6:**
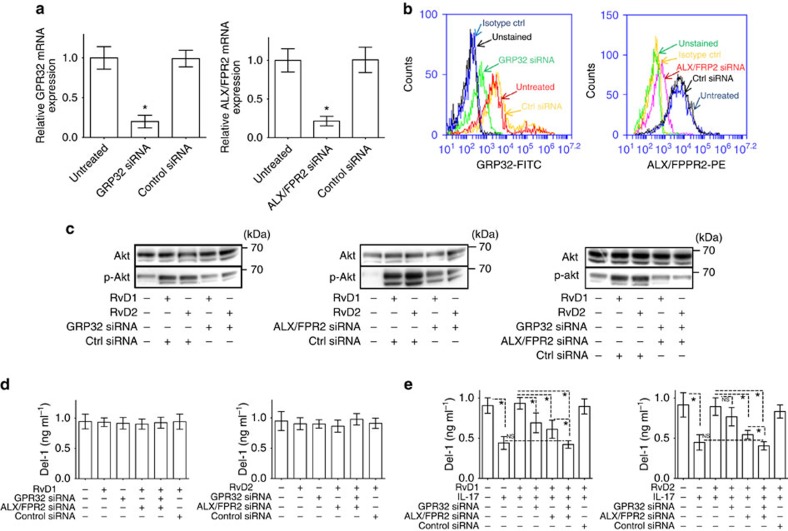
Role of GPR32 and ALX/FPR2 in the regulation of Del-1 expression in endothelial cells. (**a**,**b**) HUVEC were treated or not with siRNA to GPR32 or ALX/FPR2 or control siRNA and 24 h later were assessed for expression of these receptors at the mRNA (**a**) and protein level (**b**) by qPCR and FACS, respectively. (**c**) Akt phosphorylation (Ser473) after 15 min stimulation with 100 nM RvD1 or RvD2 in HUVEC transfected (or not) with siRNA to GPR32 and/or ALX/FPR2, as indicated. Total Akt was monitored for control purposes. (**d**) HUVEC were treated or not with siRNA to GPR32 and/or ALX/FPR2 followed by 6 h incubation with 100 nM RvD1 (left) or RvD2 (right) and assessed for Del-1 protein levels in culture supernatants by ELISA. (**e**) HUVEC transfected (or not) with siRNA to GPR32 and/or ALX/FPR2 were incubated for 6 h with 5 ng ml^−1^ IL-17 in the presence or absence of 100 nM RvD1 (left) or RvD2 (right) and assessed for Del-1 protein levels in culture supernatants by ELISA. Data are means±s.d. (*n*=5 sets of HUVEC cultures). *P<0.01 between indicated groups (ANOVA). NS, not significant.

It was previously shown that RvD1 interacts specifically with two receptors, G-protein-coupled receptor 32 (GPR32) and lipoxin A4 receptor/formyl-peptide receptor-2 (ALX/FPR2), which mediate its pro-resolution actions at least in human neutrophils and monocytes^27^,^28^. To determine whether GPR32 and/or ALX/FPR2 mediate RvD1 regulation of Del-1 in HUVEC, we knocked down the expression of these receptors with specific siRNAs (Fig. 6a,b) and determined the impact of these treatments on Akt phosphorylation and Del-1 expression. The ability of RvD1 to phosphorylate Akt was suppressed in GPR32- or ALX/FPR2-knocked down HUVEC (Fig. 6c, left and middle, respectively) and was completely abrogated in cells treated with siRNAs to both receptors (Fig. 6c, right). As expected, knocking down the expression of GPR32 or ALX/FPR2 had no significant effect on Del-1 expression in RvD1-treated HUVECs in the absence of IL-17 (Fig. 6d, left). However, knocking down GPR32 or ALX/FPR2 significantly suppressed the ability of RvD1 to reverse the inhibitory action of IL-17 on Del-1 expression (Fig. 6e, left). Knocking down both receptors at the same time resulted in complete loss of RvD1 regulation of Del-1 expression in IL-17-treated cells (Fig. 6e, left; next-to-last bar). The ability of RvD2 to phosphorylate Akt and restore the expression of Del-1 in IL-17-treated HUVEC was also affected by the same siRNA treatments, especially when both GPR32 and ALX/FPR2 were knocked down (Fig. 6c,e). Since both RvD1 and RvD2 appeared to work by a similar Akt-dependent mechanism (Figs 4, 5, 6), we next focused on RvD1 to further dissect the regulation of Del-1 expression.

### RvD1 restores C/EBPβ recruitment to the *EDIL3* promoter

**Figure 7:**
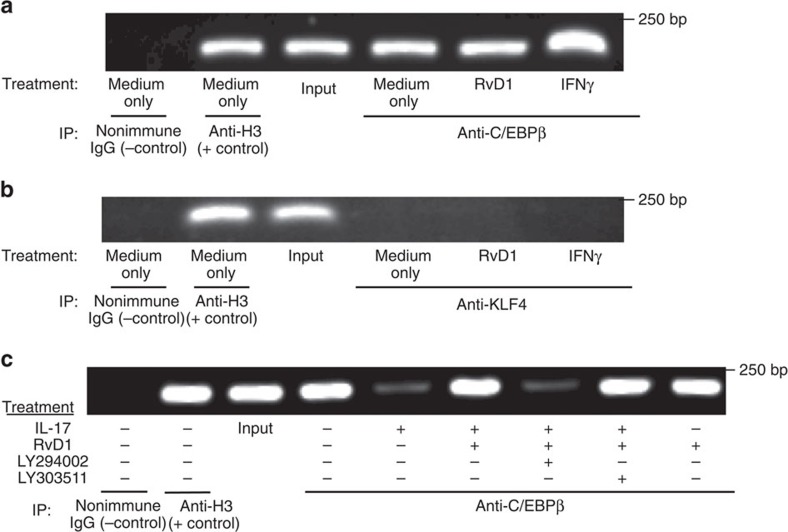
Regulation of the binding of C/EBPβ to the *EDIL3* promoter. (**a**) ChIP analysis of C/EBPβ binding to *EDIL3* promoter in HUVEC treated with medium only, RvD1 (100 nM) or IFNγ (10 ng ml^−1^). After 4 h incubation, cross-linked chromatin was immunoprecipitated with anti-C/EBPβ IgG, non-immune IgG (negative control) or with anti-Histone H3 IgG (positive control). Immunoprecipitated chromatin was subjected to PCR using specific primers designed to amplify the −328 to −589 bp region of the *EDIL3* promoter that contains C/EBPβ binding sites. PCR products were visualized by gel electrophoresis and staining with SYBR safe gel dye. (**b**) Similar experiment in which cross-linked chromatin was immunoprecipitated with anti-KLF4 IgG. (**c**) ChIP analysis of C/EBPβ binding to *EDIL3* promoter in HUVEC which were treated or not with IL-17 (10 ng ml^−1^; 4 h) with or without 30 min pre-treatment with RvD1 (100 nM). In experiments using LY249002 (20 μM; PI3K inhibitor) or LY303511 (20 μM, inactive analogue), these compounds were added 1 h before RvD1. IP, immunoprecipitation.

To strengthen the notion that RVD1 regulates Del-1 expression via an indirect manner that involves counteracting the inhibitory action of IL-17, we performed ChIP analysis. First, we showed that RvD1 treatment of HUVEC did not affect the recruitment of C/EBPβ to the *EDIL3* promoter region compared with medium only-treatment (Fig. 7a), in line with its inability to directly influence Del-1 expression (Fig. 3). In contrast, HUVEC treatment with IFNγ enhanced the recruitment of C/EBPβ to the *EDIL3* promoter region, consistent with earlier observations that IFNγ increases the transcriptional activity of C/EBPβ^19^,^20^ and directly upregulates Del-1 expression in HUVEC^21^. For control purposes, similar ChIP analysis in HUVEC that were treated or not with RVD1 or IFNγ failed to detect the presence of an irrelevant transcription factor (KLF4) at the *EDIL3* promoter region (Fig. 7b). Consistent with earlier results (Fig. 2f), IL-17 treatment of HUVEC inhibited the binding of C/EBPβ at the *EDIL3* promoter relative to untreated cells (Fig. 7c). Importantly, however, RvD1 restored the recruitment of C/EBPβ to the *EDIL3* promoter region in IL-17-treated cells; this effect was dependent on PI3K since it was inhibited by LY294002 (Fig. 7c). Taken together with the previous findings (Figs 2, 3, 4), these data show that IL-17 targets C/EBPβ through GSK-3β and leads to inhibition of C/EBPβ-dependent transcription of Del-1, although this inhibition is reversed by RvD1 in a PI3K/Akt-dependent manner.

### RvD1 reverses IL-17-induced Del-1 downregulation *in vivo*

**Figure 8:**
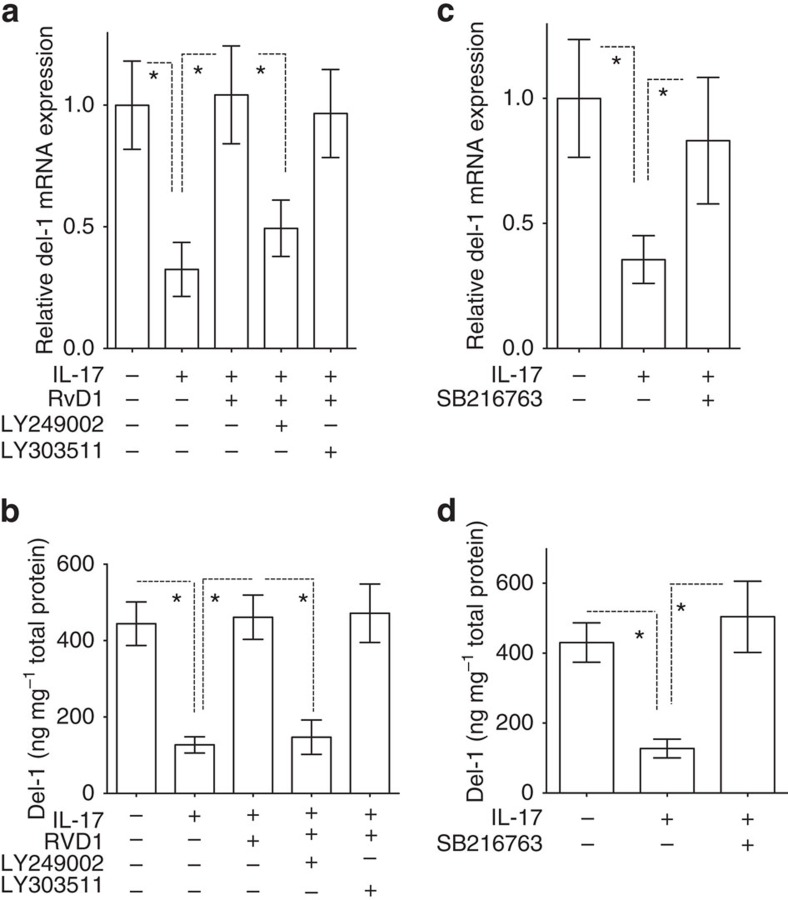
RvD1 reverses the inhibitory effect of IL-17 on Del-1 expression *in vivo*. IL-17 (1 μg) was microinjected into the palatal gingiva with or without RvD1 (1 ng) (**a**,**b**) or SB216763 (1 μg) (**c**,**d**). In (**a**,**b**) certain groups were also microinjected with LY294002 or LY303511 (0.5 μg) as indicated. Mice were euthanized after 24 h and qPCR was used to determine Del-1 mRNA expression levels in dissected gingiva (**a**,**c**), whereas ELISA of gingival tissue homogenates was performed to determine Del-1 protein levels (**b**,**d**). Different sets of mice were used for qPCR and ELISA to ensure adequate material for analysis. In qPCR, results were normalized to those of GAPDH mRNA and expressed as fold change in transcript levels relative to control mice, the average value of which was taken as 1. In ELISA, Del-1 concentrations were normalized to the total protein concentrations in the tissue homogenates. Data are means±s.d. (*n*=5 mice per experimental group and *n*=10 mice per control group; controls were treated with PBS or ethanol and gave similar results, hence the data were pooled). *P<0.01 between indicated groups (ANOVA).

The expressions of IL-17 and Del-1 are inversely correlated in both the periodontal tissue and the central nervous system in mice and humans, with Del-1 dominating in healthy and IL-17 in inflamed tissue^4^,^5^. Consistent with this, local microinjection of recombinant IL-17 into the gingiva of mice resulted in significant downregulation of Del-1 mRNA and protein expression (Fig. 8a,b, respectively). Importantly, however, the capacity of IL-17 to downregulate Del-1 was abrogated when microinjected together with RvD1 (Fig. 8a,b). RvD1, in turn, lost the ability to interfere with the IL-17 inhibition of Del-1 expression in the presence of LY294002, but not LY303511 (Fig. 8a,b). Moreover, the inhibitory effect of IL-17 on Del-1 mRNA and protein expression was blocked when the cytokine was microinjected together with SB216763 (Fig. 8c,d). These data suggest that the ability of IL-17 to inhibit Del-1 *in vivo* is differentially regulated by PI3K and GSK-3β in negative and positive manner, respectively.

**Figure 9:**
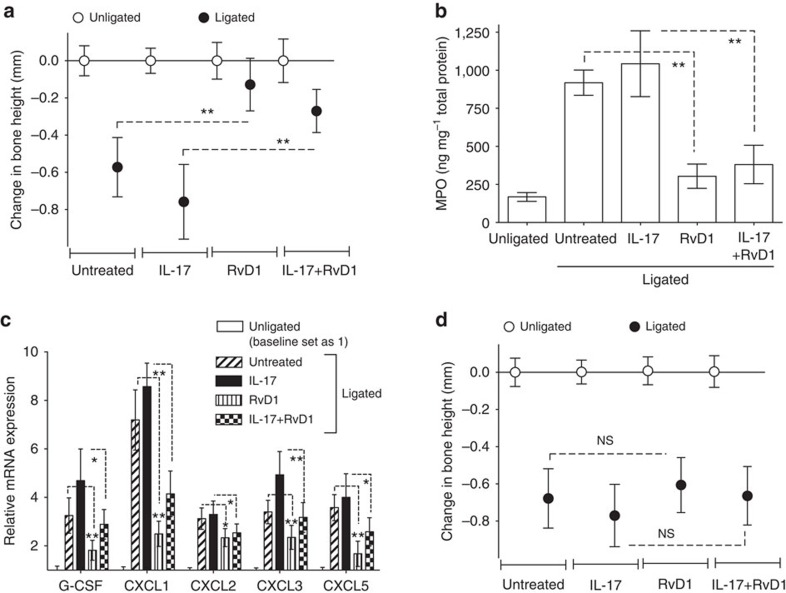
RvD1 inhibits ligature-induced bone loss. Periodontal bone loss was induced for 5 days in wild-type (**a**–**c**) or Del-1-deficient (**d**) mice by ligating a maxillary second molar and leaving the contralateral tooth unligated (baseline control). The mice were locally microinjected into the palatal gingiva with IL-17 (0.2 μg), RvD1 (0.2 ng), or their combination, 1 day before placing the ligature and every day thereafter until the day before sacrifice (day 5). Ligature-induced periodontal inflammation in wild-type mice was monitored in dissected gingival processed for determining MPO (a measure of neutrophil infiltration) by ELISA (**b**) or for qPCR to determine mRNA expression of the indicated molecules, normalized against GAPDH mRNA (**c**). The data are expressed as fold change in the transcript abundance in the ligated side relative to that of the unligated side, which was assigned an average value of 1. Data are means±s.d. (*n*=5 mice). *P<0.05 and **P<0.01 between indicated groups (unpaired *t*-test).

We next examined whether RvD1 can also regulate disease activity in a murine model of ligature-induced periodontitis, where the resulting bone loss is IL-17−dependent (the model induces high levels of gingival IL-17 expression, and bone loss can be inhibited by local anti-IL-17 treatment^4^,^29^). To this end, we microinjected RvD1 in the gingiva of wild-type or Del-1-deficient mice subjected to ligature-induced periodontitis, either alone or together with recombinant IL-17. In wild-type mice, RvD1 significantly inhibited periodontal bone loss by 78% and 64%, depending on whether it was administered alone or together with IL-17, respectively (Fig. 9a). RvD1-mediated inhibition of bone loss was associated with significantly reduced levels of myeloperoxidase (MPO; a quantitative marker of neutrophil infiltration) (Fig. 9b) and decreased mRNA expression of neutrophil-associated molecules, such as G-CSF and several CXC chemokines (Fig. 9c) in the gingival tissue. Intriguingly, RvD1 failed to significantly inhibit bone loss in Del-1-deficient mice (Fig. 9d), suggesting that—at least in this model—RvD1 promotes tissue homeostasis in a manner that is heavily dependent on Del-1.

## Discussion

**Figure 10:**
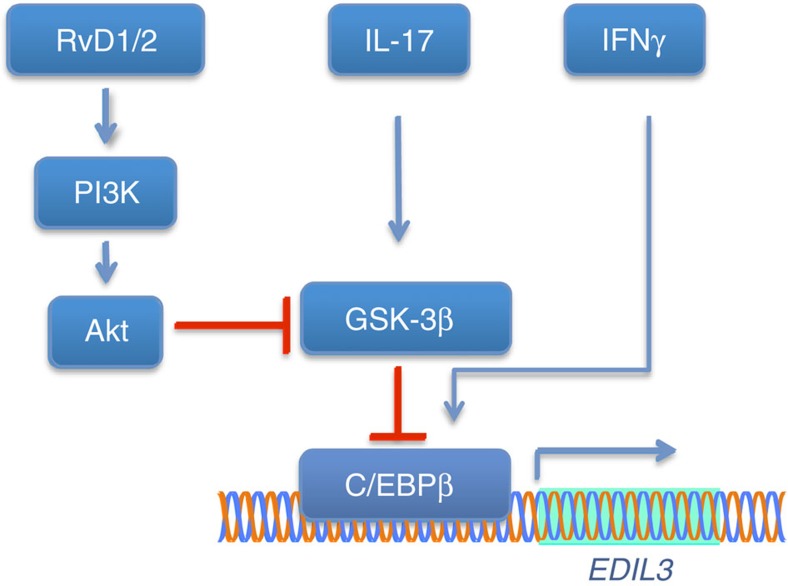
Model of Del-1 regulation. IL-17 downregulates Del-1 expression through a GSK3β- and C/EBPβ-dependent mechanism, which inhibits the binding of the critical transcription factor C/EBPβ to the *EDIL3* promoter. This inhibitory action of IL-17 is counteracted at the GSK3β level by D-series resolvins in a PI3K/Akt-dependent manner. In stark contrast to IL-17, IFNγ enhances the binding of C/EBPβ to the *EDIL3* promoter and upregulates Del-1 expression.

Del-1 has recently emerged as an endothelial cell-secreted homeostatic factor that regulates local inflammation in several tissues^3^,^4^,^5^,^6^,^7^. To our knowledge, this is the first comprehensive effort to understand the regulation of Del-1 expression, leading to the identification of crucial regulatory factors (ligands, kinases and transcription factors) some of which can be exploited as therapeutic targets to promote Del-1-mediated tissue homeostasis. Although IL-17 induces the expression of numerous target genes via well-characterized signalling pathways, relatively little is known about inhibitory signalling events mediated by IL-17 (refs 11, 30). It was thus important to define the signalling pathway by which IL-17 inhibits Del-1 expression. In this report, we provide *in vitro* and *in vivo* evidence that IL-17 inhibits the expression of Del-1 by acting via a GSK-3β-dependent pathway. This inhibitory effect of IL-17 is abrogated by D-series resolvins at the GSK-3β level in a PI3K/Akt-dependent manner (Fig. 10).

We have moreover implicated C/EBPβ as a critical transcription factor in Del-1 expression using independent approaches (Del-1 mRNA and protein expression analysis following silencing or overexpression of C/EBPβ or upstream signalling molecules, ChIP analysis, and reporter gene assays). The capacity of the GSK-3β inhibitor, SB216763, to restore the binding of C/EBPβ to the *EDIL3* promoter in IL-17-treated HUVEC was associated with inhibition of C/EBPβ phosphorylation. This finding is consistent with earlier reports that phosphorylation of C/EBPβ by GSK-3β inhibits its DNA-binding capacity and transcription of target genes^10^,^23^. Intriguingly, whereas IL-17 phosphorylates and inhibits the recruitment of C/EBPβ to the *EDIL3* promoter region in a GSK-3β-dependent manner, D-series resolvins interfere with this mechanism. In this regard, we have shown that Akt is critical for the ability of RvD1 and RvD2 to induce Ser9 phosphorylation of GSK-3β, thereby abrogating its inhibitory phosphorylation of C/EBPβ and allowing unhindered expression of Del-1.

Among several stimuli we have recently tested, IFNγ uniquely upregulated the expression of Del-1 (ref. 21). In the present study, we have shown that IFNγ enhances the recruitment of C/EBPβ to the *EDIL3* promoter region, in stark contrast to the inhibitory effect of IL-17. Given the pro-inflammatory role of IFNγ in several experimental systems^31^, its ability to upregulate Del-1 might appear paradoxical. However, it should be noted that IFNγ also has regulatory functions, which include inhibition of Th17 differentiation^32^,^33^ and of osteoclastogenesis^34^. The latter provides another example in which IFNγ and IL-17 mediate differential effects. Indeed, whereas IL-17 upregulates RANKL expression and promotes osteoclastogenesis, IFNγ inhibits RANKL-induced osteoclastogenesis^34^,^35^.

In contrast to Del-1, which is produced by endothelial cells^3^,^4^, pro-resolving lipid mediators are generated at the endothelial leukocyte interface through transcellular biosynthesis^36^. In this regard, endothelial cells act as donor cells that convert a precursor lipid compound into an intermediate product that is subsequently converted into the final active lipid compound by activated enzymes in neutrophils, hence acting as acceptor cells^36^. Therefore, when released within the vascular lumen, D-series resolvins become available to interfere with the inhibitory effect of IL-17 on endothelial cell expression of Del-1. In the context of the homeostatic functions of Del-1 (refs 4, 5), the ability of RvD1 (and RvD2) to reverse IL-17-induced Del-1 downregulation constitutes a novel mechanism in their pro-resolution program, which further includes clearance of inflammatory debris, tissue regeneration and promotion of antimicrobial defenses^13^,^16^. In the current study, the capacity of RvD1 to block IL-17-driven bone loss was vitally dependent on Del-1, further supporting the notion for the operation of a protective RvD1*−*Del-1 axis against IL-17-induced pathologic inflammation. In counter-regulating IL-17 inhibition of Del-1 expression in endothelial cells, RvD1 appeared to use the same receptors (GPR32 and ALX/FPR2) it uses in neutrophils and monocytes^27^,^28^.

Although Del-1 contributes crucially to the capacity of the periodontal tissue to self-regulate and prevent persistent inflammation, this local homoeostatic mechanism breaks down in aged mice (⩾18 months old) due to age-related decline in Del-1 mRNA and protein expression^4^,^21^. The mechanism, however, underlying the age-associated downregulation of Del-1 expression remains uncertain. Interestingly in this context, it was recently shown that old mice have significantly reduced levels of pro-resolving lipid mediators, including D series resolvins such as RvD1, as compared with the levels of these compounds in young mice^37^. The relative scarcity of resolvins in old mice might therefore contribute to the age-related decline of Del-1 expression. Alternatively, or in addition, the downregulation of Del-1 expression might be caused by the elevated expression of IL-17 seen in old mice^4^. Since recombinant Del-1 administration blocks periodontal inflammation and bone loss in old mice, approaches that restore the expression of Del-1 in the elderly could find application for the treatment of age-associated inflammatory diseases including periodontitis. This prevalent oral inflammatory disease is not only a common cause of tooth loss but is also associated with increased risk for certain systemic conditions, such as atherosclerosis, rheumatoid arthritis and adverse pregnancy outcomes^38^. The health impact of periodontitis and its economic burden require novel, host-modulation treatments to complement conventional mechanical therapy, which often is not sufficient by itself to control the disease^16^,^39^,^40^.

Besides acting as a gatekeeper of inflammation in the periodontal tissue and the central nervous system^4^,^5^, Del-1 was also shown to play a protective role against inflammation-mediated adrenal gland dysfunction^6^, salivary gland inflammation in Sjögren's syndrome^41^, and pulmonary fibrosis^7^. In all these conditions, Del-1 expression levels are diminished during disease^6^,^7^,^41^. Moreover, the Del-1-encoding gene (*EDIL3*) has been identified as a disease susceptibility gene in multiple sclerosis^42^, Alzheimer's disease^43^, and ankylosing spondylitis^44^. The identification of factors, such as resolvins, that restore Del-1 expression is therefore of potential importance in at least these inflammatory conditions.

In summary, *in vitro* and *in vivo* evidence generated in this study shows that IL-17 downregulates Del-1 expression through a GSK-3β- and C/EBPβ-dependent mechanism, whereas D-series resolvins intercept and block this inhibitory pathway at the GSK-3β level by activating PI3K/Akt signalling (Fig. 10). The differential effects of IL-17 and RvD1 on Del-1 expression can be exploited to regulate Del-1 in desired directions depending on context, that is, downregulation in acute infections and upregulation in chronic inflammatory disorders.

## Methods

### Reagents

Recombinant human or mouse IL-17A was purchased from R&D Systems. The D series resolvins, RvD1 and RvD2, and MK2206 (Akt inhibitor) were from Cayman Chemical. SB202190 (p38 MAP kinase inhibitor), SB216763 (GSK-3β inhibitor), SN50 (NF-κB inhibitor), SP600125 (JNK inhibitor) were obtained from EMD-Millipore. LY294002 (PI3K inhibitor) and LY30351 (inactive analogue of LY294002) were purchased from Sigma-Aldrich. Rabbit polyclonal antibody to Del-1 was from ProteinTech. Rabbit monoclonal antibody (mAb) to β-actin (13E5), rabbit IgG antibodies to total Akt (phosphorylation state independent), phospho-Akt (Ser473), total GSK-3β and phospho-GSK-3β (Ser9), as well as rabbit anti-phospho-C/EBPβ (Thr-188) and anti-phospho-threonine IgG antibodies were purchased from Cell Signaling Technology. Rabbit IgG antibodies to GPR32 and to ALX/FPR2 were from Abcam and Abnova, respectively. FITC- or PE-conjugated goat anti-rabbit IgG and rabbit IgG antibody to C/EBPβ were purchased from Santa Cruz Biotechnology. All reagents (including those mentioned in the description of assays below) were used at doses based on our previously published work or on experiments in this study (see Supplementary Figs 1 and 4).

### Cell culture

HUVEC (Lonza C2517A) were seeded in 12-well culture plates in complete EBM-2 medium (Lonza) and grown to confluence in a humidified atmosphere at 37 °C and 5% CO2. The cells were treated as outlined in the figure legends and were subjected to several assays as described below.

### Quantitative real-time PCR

Total RNA was extracted from dissected gingival tissue or from cultured HUVEC using the PerfectPure RNA cell kit (5 Prime, Fisher) and quantified by spectrometry at 260 and 280 nm. The RNA was reverse-transcribed using the High-Capacity cDNA Archive kit (Applied Biosystems) and real-time PCR with cDNA was performed using the ABI 7500 Fast System, according to the manufacturer's protocol (Applied Biosystems). Data were analysed using the comparative (ΔΔCt) method. TaqMan probes, sense primers, and antisense primers for detection and quantification of genes investigated in this paper were purchased from Applied Biosystems/Life Technologies.

### Immunoprecipitation and immunoblotting

Cell lysates were prepared using the RIPA Lysis Buffer System (Santa Cruz Biotechnology) and protein content concentrations were determined using the Bio-Rad Bradford protein assay. Immunoprecipitation was carried out using rabbit IgG to C/EBPβ and protein G-coated immunomagnetic beads according to the manufacturer's protocol (Active Motif; Universal Magnetic Co-IP kit). Proteins were separated by standard SDS with polyacrylamide gel electrophoresis on 10% acrylamide gels (Life Technologies) and transferred to polyvinylidene difluoride membrane (Bio-Rad) by electroblotting. The membranes were incubated in blocking buffer (5% nonfat dried milk, 10 mM Tris (pH 7.5), 100 mM NaCl, and 0.05%Tween 20) followed by probing with primary antibodies and visualization with horseradish peroxidase-conjugated secondary antibody and chemiluminescence using the Amersham Biosciences ECL system. Images were captured using a FluorChem M imaging system (ProteinSimple). The sources of the various antibodies used are listed above under Reagents. For immunoblotting, the various antibodies were used at the following dilutions: Rabbit mAb (13B5) to β-actin (1:3000); rabbit IgG to total Akt (1:3000); rabbit IgG to phospho-Akt (Ser473) (1:3000); rabbit IgG to total GSK-3β (1:3000); rabbit IgG to phospho-GSK-3β (Ser9) (1:3000); rabbit IgG to total C/EBPβ (1:500); rabbit IgG to phospho-C/EBPβ (Thr-188) (1:2000); and rabbit IgG to phospho-threonine (1:2000). For immunoprecipitation, rabbit IgG to C/EBPβ was used at a dilution of 1:50.

### Del-1 ELISA

A sandwich ELISA for Del-1 was performed as previously reported^45^. Specifically, the levels of Del-1 in HUVEC culture supernatants were determined on microtiter plates coated with capture antibody (mouse anti-human Del-1 mAb; clone D345, IgG1—kindly provided by Dr Perumal Thiagarajan) and developed using polyclonal rabbit IgG antibody to human Del-1 (prepared by Covance Research Products) followed by peroxidase-conjugated goat anti-rabbit IgG (R&D Systems) and tetramethyl benzidine chromogenic substrate. Optical density values were measured in a microplate reader (Biotek) and the assay was calibrated by means of a serially diluted Del-1 protein standard.

### MPO assay

The concentration of MPO in gingival tissue homogenates was determined using an ELISA kit according to the manufacturer’s instructions (Hycult Biotechnology). MPO concentrations were normalized to the total protein concentrations in the tissue homogenates, as measured using the Coomassie Plus Bradford protein assay kit (Pierce).

### Chromatin immunoprecipitation

ChIP analysis of C/EBPβ binding to *EDIL3* promoter in HUVEC was performed using the SimpleChIP Plus Enzymatic Chromatin IP Kit with magnetic beads (Cell Signaling). Cross-linked chromatin prepared from HUVEC treated as described in the legend to Fig. 7 was immunoprecipitated with rabbit IgG antibody to C/EBPβ (C19 sc-150; Santa Cruz). ChIP with non-immune rabbit IgG or with rabbit IgG mAb to anti-Histone H3 (D2B12; Cell Signaling) was used as negative and positive control, respectively. Primers flanking a C/EBPβ binding site in the *EDIL3* promoter (−328 to −589 bp) were used for PCR (5′- CTT ATA GCA GAA GGA GCT GAA AGA G -3′ and 5′- TGG AGA ACA ATG AAG GCG TGA G -3′). PCR was performed using immunoprecipitated chromatin and PCR products were visualized by 2% agarose gel electrophoresis and staining with SYBR safe gel dye. For control purposes, a similar procedure was followed for ChIP analysis of KLF4 binding to the same region of the *EDIL3* promoter using rabbit IgG antibody to KLF4 (H-180 sc-20691; Santa Cruz).

### RNA interference and gene overexpression

siRNAs against C/EBPβ, Akt, GPR32 and ALX/FPR2 were synthesized by Ambion. For transient transfections of siRNA, the sense and antisense oligoribonucleotides were annealed at a concentration of 20 nM and transfected into HUVEC in six-well plates using the Lipofectamine RNAiMAX reagent, according to the manufacturer’s protocol (Life Technologies). The Ambion’s silencer negative control siRNA was used to confirm that the transfection did not cause nonspecific effects on gene expression. Knockdown expression of C/EBPβ and Akt at the protein level was confirmed by immunoblotting (see above). Knockdown expression of GPR32 and ALX/FPR2 at the protein level was confirmed by FACS on a BD Accuri C6 flow cytometer after staining the cells with rabbit IgG antibodies to GPR32 (Abcam; diluted 1:250) and to ALX/FPR2 (Abnova; diluted 1:250) followed by FITC- or PE-conjugated goat anti-rabbit IgG (diluted 1:500). C/EBPβ or GSK-3β expression vectors were constructed by inserting cDNA encoding human C/EBPβ or GSK-3β into the EcoRI-XhoI site or the Sgf1-Mlu1 site, respectively, of the pCMV6-ENTRY vector (Origene). HUVEC were transfected with the C/EBPβ or GSK-3β expression vector or empty vector using TransPass HUVEC Transfection Reagent (New England Biolabs) following the manufacturer’s recommended protocol.

### Luciferase reporter assay

To construct a human *EDIL3* promoter/luciferase reporter plasmid (hEDIL3-promoter-Luc), a −168/+20 promoter fragment (which contains two C/EBPβ binding sites) was amplified by PCR from human genomic DNA using the primers 5′- CCT CTA CTA AAT AAT GTG TC -3′ and 5′- AGC CCT GGT TGG CTG GGC G -3′. The amplification product was subcloned into the *Mlu1-Bgl2* sites of the promoterless pLightSwitch_Prom luciferase reporter vector (Active motif) and confirmed by sequencing. For luciferase assays, 1 × 10^5^ HEK-293T cells (ATCC CRL-3216) were seeded on 96-well plates and co-transfected with hEDIL3-promoter-Luc and pGL3 firefly luciferase reporter plasmid (Promega) as an internal transfection control, using FuGENE HD transfection reagent (Promega). In experiments involving siRNA treatment, the cells were co-transfected with the above-described plasmids and siRNA (20 nM) using DharmaFECT Duo (GE Healthcare). Luciferase assays were performed using the Britelite Assay System (Perkin-Elmer)^46^. In experiments with IL-17, HEK-293T cells were additionally transiently co-transfected with IL-17RA and IL-17RC (Origene) to render them responsive to IL-17 (HEK-293T-IL-17RA/C cells)^47^.

### Colorimetric phospho-AKT assay

The activation of AKT (Ser473 phosphorylation) was determined using a colorimetric cell-based ELISA kit according to the protocol of the manufacturer (Active Motif). The assay is based on an antibody specific for Ser473-phosphorylated AKT and total AKT is determined using an antibody that recognizes AKT regardless of its phosphorylation state.

### Mice and ligature-induced periodontitis

All animal procedures were approved by the University of Pennsylvania Institutional Animal Care and Use Committee, in compliance with established federal and state policies. Female C57BL/6J mice, 8- to 10-week old, were used in the experiments. The generation of C57BL/6J *Edil3*^−/−^ mice has been described^3^. Wild-type C57BL/6J mice were purchased from the Jackson Laboratories. Experiments were performed using randomly assigned mice and no animals were withdrawn from the studies, according to predetermined criteria in the IACUC protocols, or were excluded from any of the analyses. The experiments involved five mice per group (determined by GraphPad StatMate power analysis for *P*=0.05 and a power of 0.80). To determine whether RvD1 counteracts the inhibitory effect of IL-17 on Del-1 expression *in vivo*, IL-17 was microinjected into the palatal gingiva with or without RvD1 and signalling inhibitors at doses specified in the legend to Fig. 6. The mice were killed 24 h later and dissected gingiva were processed for quantitative real-time PCR (qPCR) determination of Del-1 mRNA expression levels, as described above (see Quantitative real-time PCR).

The placement of ligatures accelerates bacteria-mediated inflammation and bone loss^48^. To this end, a 5-0 silk ligature was tied around the maxillary left second molar, whereas the contralateral molar tooth in each mouse was left unligated to serve as baseline control in the bone loss measurements^49^. The mice were euthanized five days after placement of the ligatures. Periodontal bone loss was assessed morphometrically in defleshed maxillae using a dissecting microscope (× 40) fitted with a video image marker measurement system (Nikon Instruments). Specifically, the distance from the cement-enamel junction (CEJ) to the alveolar bone crest (ABC) was measured on predetermined points on the ligated second molar (three sites corresponding to mesio-palatal cusp, palatal groove, and disto-palatal cusp) and the affected adjacent regions (sites corresponding to disto-palatal groove and distal cusp of the first molar, and palatal cusp of the third molar)^49^. To calculate bone loss, the 6-site total CEJ-ABC distance for the ligated side of each mouse was subtracted from the 6-site total CEJ-ABC distance of the contralateral unligated side. The results were presented in mm and negative values indicated bone loss relative to the baseline (unligated control). In intervention experiments, RvD1 with or without IL-17 (see legend to Figs 8 and 9 for details) was microinjected into the palatal gingiva of the ligated second maxillary molar using a 33-gauge stainless steel needle attached to a micro syringe (Hamilton)^4^,^29^.

### Statistical analysis

Data were evaluated by one-way ANOVA and the Dunnett’s multiple-comparison test using the InStat program (GraphPad). Where appropriate (comparison of two groups only), two-tailed unpaired *t*-tests were performed. Data shown are representative of two or more independent experiments. A *P*-value<0.05 was taken as the level of significance.

## Additional information

**How to cite this article:** Maekawa, T. *et al*. Antagonistic effects of IL-17 and D-resolvins on endothelial Del-1 expression through a GSK-3β-C/EBPβ pathway. *Nat. Commun.* 6:8272 doi: 10.1038/ncomms9272 (2015).

## Supplementary Material

Supplementary Information Supplementary Figures 1-6
