# Supplementary material for: Antagonistic effects of IL-17 and D-resolvins on endothelial Del-1 expression through a GSK-3β-C/EBPβ pathway
Source: Nat Commun. 2015 Sep 16;6:8272. doi: 10.1038/ncomms9272 (PMC4573473; doi:10.1038/ncomms9272)
Supplement: Supplementary Information — Supplementary Figures 1-6 [file ncomms9272-s1.pdf]

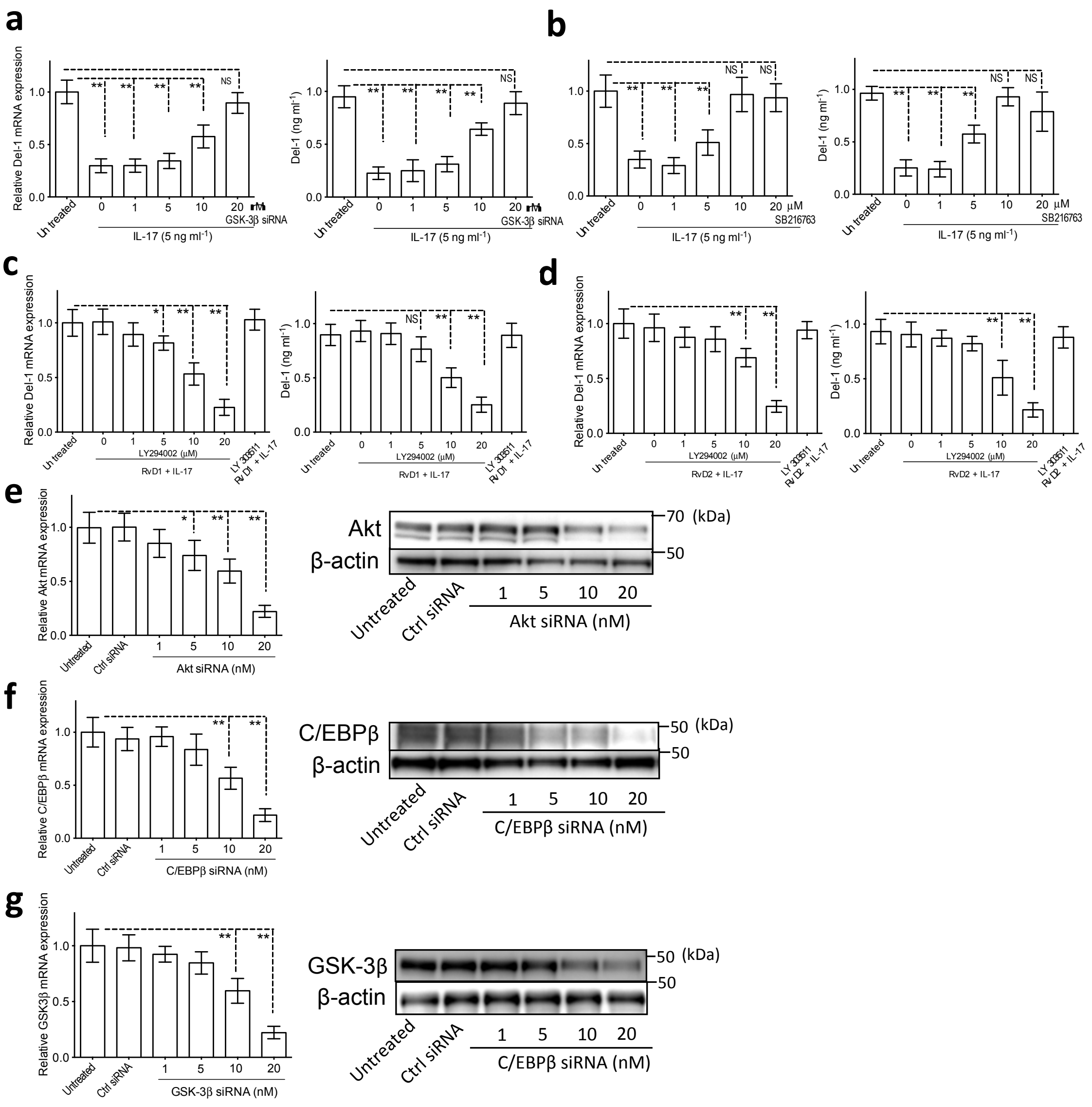

Supplementary figure 1 | Dose-response experiments of inhibitors used in the study. (a-b) HUVEC were treated with the indicated concentrations of siRNA to GSK-3β (**a**) or of SB216763 (**b**) followed by stimulation with IL-17 (5 ng ml^{-1}) for 2h to determine Del-1 mRNA expression by qPCR (left panels) or 6h to determine Del-1 protein levels in culture supernatants by ELISA (right panels). (**c**) HUVEC were pretreated for 1h with the indicated concentrations of LY249002 or its inactive analog LY303511 (20 μM) and then incubated with RVD1 (100 nM) and IL-17 (5 ng ml^{-1}) for 2h to determine Del-1 mRNA expression by qPCR (left panel) or 6h to determine Del-1 protein levels in culture supernatants by ELISA (right panel). Note that in the absence of LY249002 (0 μM), the expression of Del-1 is similar between untreated HUVEC and HUVEC exposed to both RvD1 and IL-17 because RvD1 completely abrogates the inhibitory effect of IL-17 on Del-1 expression (see Fig. 4a). (**d**) Similar experiment as in **c** using RvD2 in lieu of RvD1. (**e-g**) HUVEC were treated with control (ctrl) siRNA or specific siRNA to Akt (**b**), C/EBPβ (**c**), or GSK-3β (**d**) at the indicated concentrations and after 24h the expression of these molecules was determined at the mRNA level by qPCR (left panels) or at the protein level by immunoblotting (right panels; β-actin was used as control). Data are means ± SD (n = 5 sets of HUVEC cultures). *$P < 0.05$ and **$P < 0.01$ between indicated groups (ANOVA). NS, not significant.

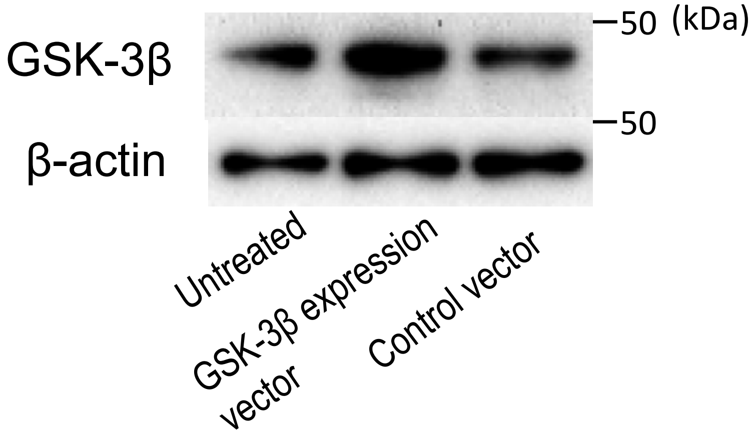

Supplementary figure 2 | Overexpression of GSK-3β in HUVEC. HUVEC were transfected with control vector or with GSK-3β expression vector. After 24h, the cells were lysed and whole-cell-lysate immunoblotting with specific antibodies was used to monitor the levels of GSK-3β and β-actin (control).

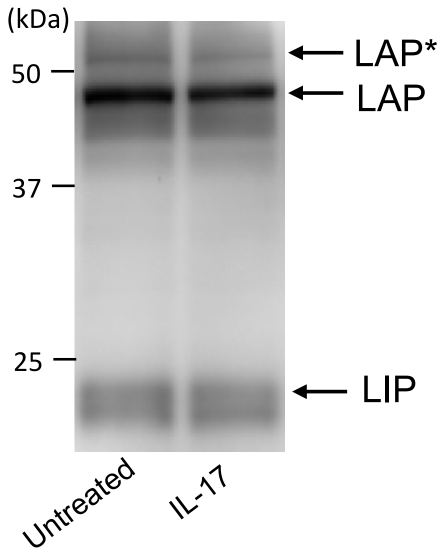

Supplementary figure 3 | HUVEC were treated or not with IL-17 (5 ng ml^{-1}) and the expression of C/EBPβ isoforms (LAP*, LAP, and LIP) was examined by immunoblotting with an antibody that can detect all three isoforms.

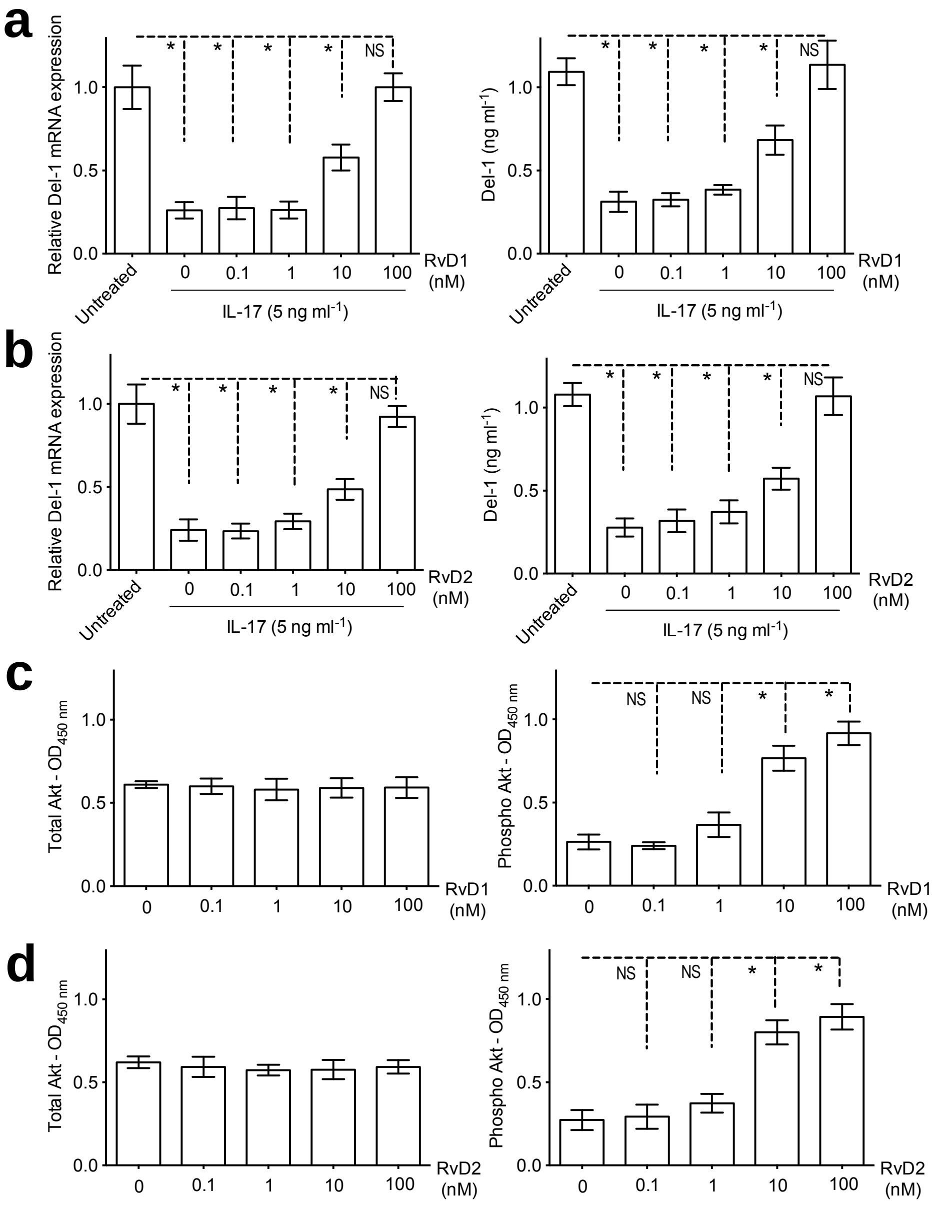

Supplementary figure 4 | RvD1 and RvD2 phosphorylate Akt and counteract IL-17 inhibition of Del-1 expression: Dose-response experiments. (**a**, **b**) HUVEC were pretreated for 30 min with RvD1 (**a**) or RvD2 (**b**) at the indicated concentrations and then incubated with IL-17 (5 ng ml^{-1}) for 2h to determine Del-1 mRNA expression by qPCR (left panels) or 6h to determine Del-1 protein levels in culture supernatants by ELISA (right panels). (**c**, **d**) HUVEC were treated with RvD1 or RvD2 at the indicated concentrations for 15 minutes and fixed. Total (left panels) and phospho-AKT (right panels) were determined in using a colorimetric cell-based ELISA kit based on antibodies specific for Ser473-phosphorylated AKT and total AKT regardless of its phosphorylation state (Active Motif). Data are means ± SD (**a,b** *n* = 5 and **c,d** *n* = 3 sets of HUVEC cultures). *P < 0.01 between indicated groups (ANOVA). NS, not significant.

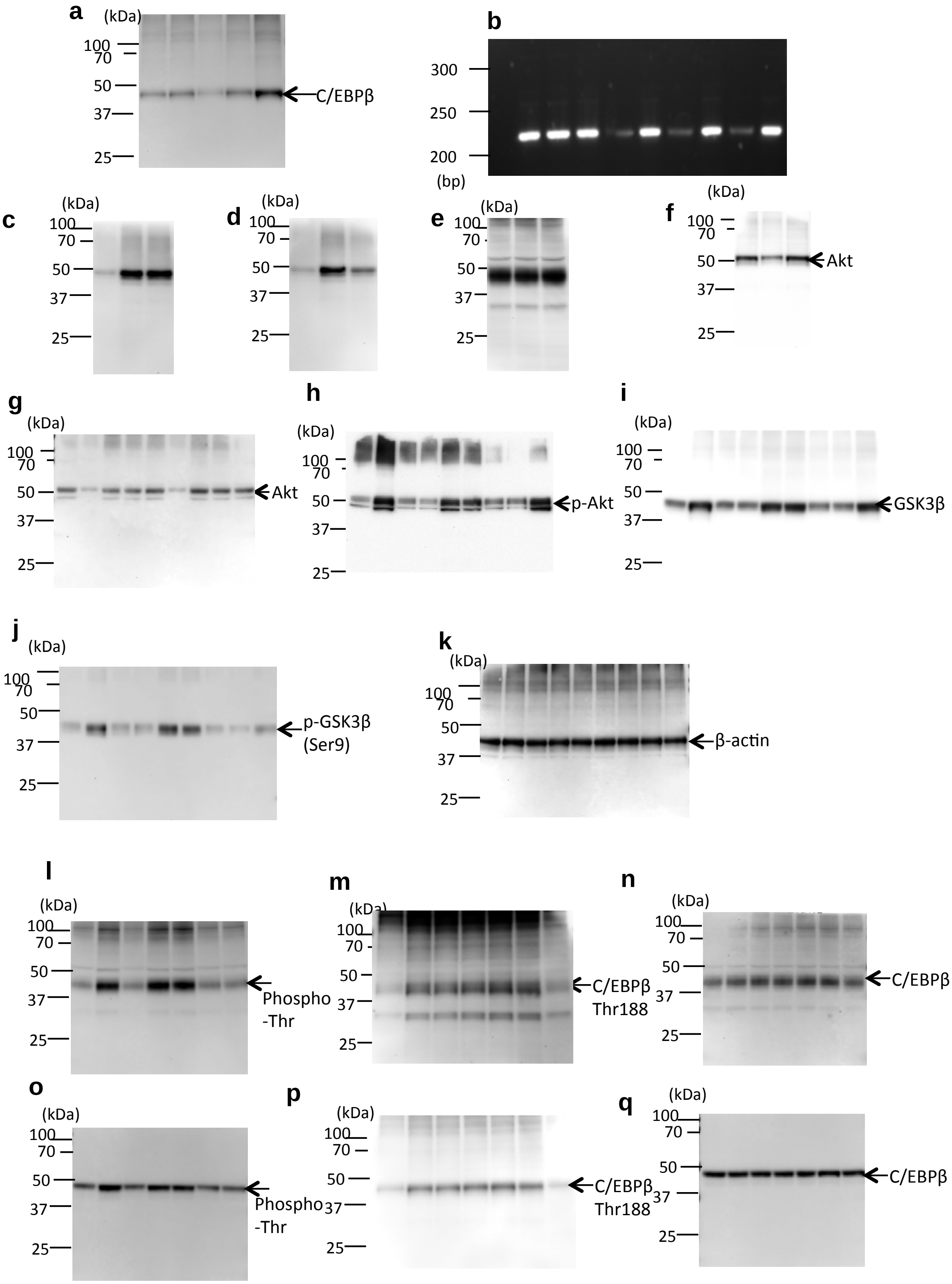

a
(kDa)
100
70
50
37
25
C/EBPβ
b
300
250
200
(bp)
c
(kDa)
100
70
50
37
25
d
(kDa)
100
70
50
37
25
e
(kDa)
100
70
50
37
25
f
(kDa)
100
70
50
37
25
Akt
g
(kDa)
100
70
50
37
25
Akt
h
(kDa)
100
70
50
37
25
p-Akt
i
(kDa)
100
70
50
37
25
GSK3β
j
(kDa)
100
70
50
37
25
p-GSK3β
(Ser9)
k
(kDa)
100
70
50
37
25
β-actin
l
(kDa)
100
70
50
37
25
Phospho
-Thr
m
(kDa)
100
70
50
37
25
C/EBPβ
Thr188
n
(kDa)
100
70
50
37
25
C/EBPβ
o
(kDa)
100
70
50
37
25
Phospho
-Thr
p
(kDa)
100
70
50
37
25
C/EBPβ
Thr188
q
(kDa)
100
70
50
37
25
C/EBPβ

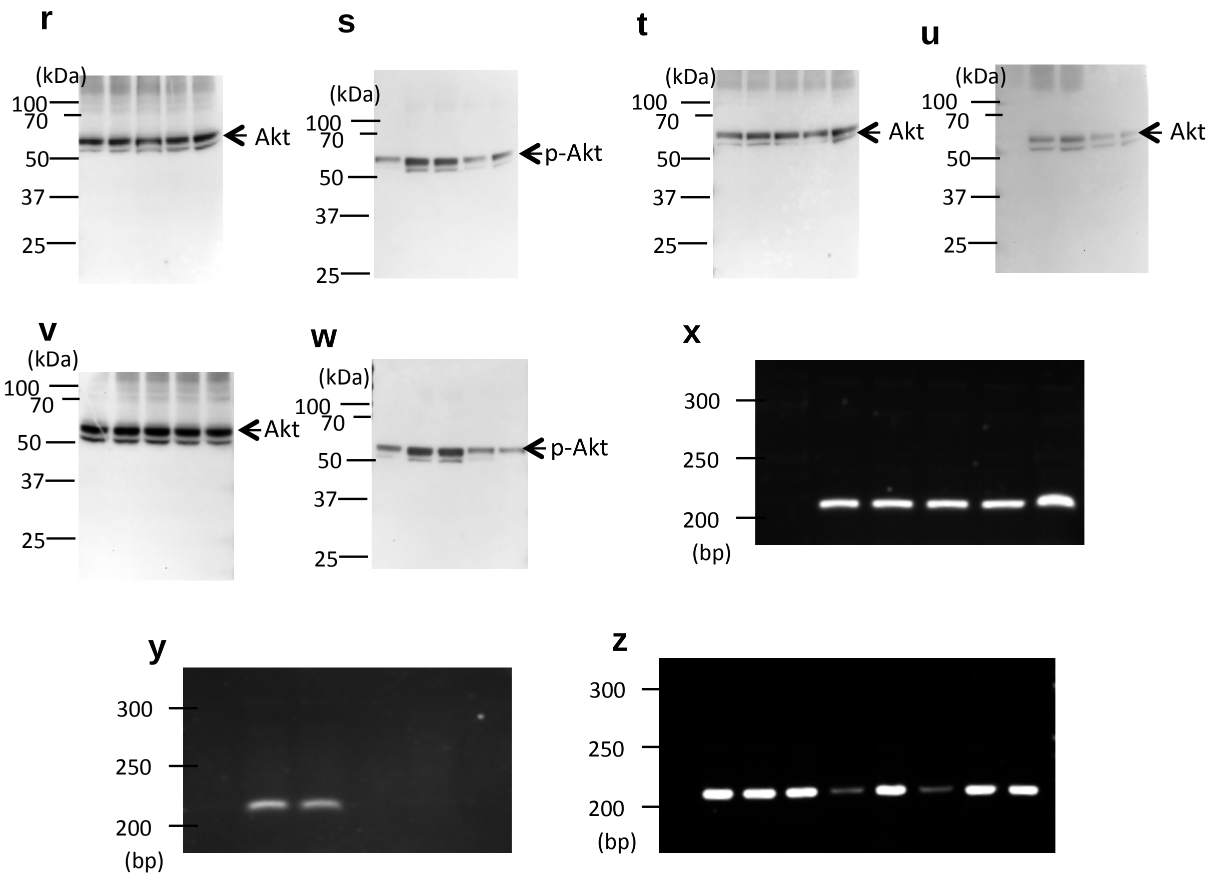

Supplementary figure 5 | Uncropped scans of immunoblots or gels for the main figures. (a) Fig. 2a - C/EBP; **(b)** Fig. 2f; **(c)** Fig. 2g - IB:phosphor-Thr; **(d)** Fig. 2g - IB:C/EBPβ(Thr188); **(e)** Fig. 2g - IB:C/EBPβ; **(f)** Fig. 4c - Akt; **(g)** Fig. 5a - Akt **(h)** Fig. 5a - p-Akt(Ser473); **(i)** Fig. 5a - GSK3β; **(j)** Fig. 5a - p-GSK3β(Ser9); **(k)** Fig. 5a - β-actin; **(l)** Fig. 5b - IB:phosphor-Thr; **(m)** Fig. 5b - IB:C/EBPβ Thr188; **(n)** Fig. 5b - IB:C/EBPβ; **(o)** Fig. 5c - IB:phosphor-Thr; **(p)** Fig. 5c - IB:C/EBPβ(Thr188); **(q)** Fig. 5c - IB:C/EBPβ; (**r**) Fig. 6c(left) - Akt; (**s**) Fig. 6c(left) - p-Akt(Ser473); (**t**) Fig. 6c (middle) - Akt; (**u**) Fig. 6c (middle) - p-Akt(Ser473); (**v**) Fig. 6c (right) – Akt; (**w**) Fig. 6c (right) - p-Akt(Ser473); (**x**) Fig. 7a; (**y**) Fig. 7b; (**z**) Fig. 7c.

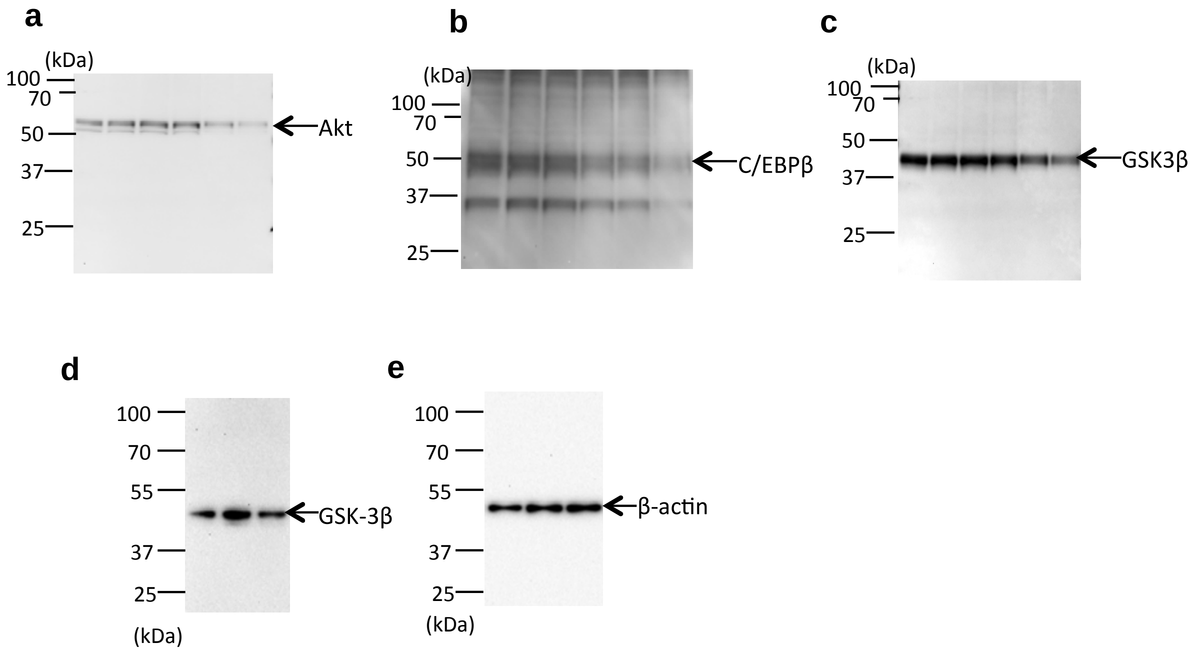

Supplementary figure 6 | Uncropped scans of immunoblots for supplementary figures. (a) Supplementary fig. 1e – Akt; **(b)** Supplementary fig. 1f - C/EBPβ; **(c)** Supplementary fig. 1g - GSK3β; **(d)** Supplementary fig. 2 – GSK-3β; **(e)** Supplementary fig. 2 - β-actin.
